# AFRICAN-AMERICAN DEMENTIA CAREGIVER STRATEGIES INVENTORY (DCSI-A): INITIAL PSYCHOMETRIC EVALUATION

**DOI:** 10.1093/geroni/igac059.3067

**Published:** 2022-12-20

**Authors:** Nik Lampe, Robert Glueckauf, Benjamin Behers, Yuxia Wang, Sarai Jean-Pierre

**Affiliations:** Vanderbilt University, Nashville, Tennessee, United States; Florida State University College of Medicine, Tallahassee, Florida, United States; Florida State University College of Medicine, Tallahassee, Florida, United States; Florida State University College of Medicine, Tallahassee, Florida, United States; Florida State University College of Medicine, Tallahassee, Florida, United States

## Abstract

Recent studies have found promising evidence of the benefits of cognitive-behavioral interventions (CBIs) in improving depression and health status of African-American dementia caregivers (CGs). However, limited information exists about the specific CB strategies used in these investigations and which CB strategies were most effective in reducing caregiver distress and distress-related health problems. To address these shortcomings, Glueckauf and colleagues developed the African-American Dementia Caregiver Strategies Inventory (DCSI-A) using data from two CBI interventions (Nf129). The primary objectives of the current study were: (a) to identify the most frequently used categories of CBI strategies deployed by African-American CGs and (b) to conduct an initial evaluation of the psychometric properties of the DCSI-A. Descriptive statistics were calculated to assess strategy category frequency and percent of total responses. The two most frequently used strategies were: (1) CG Health-Related Intervention Strategies with 19 endorsements, 16.10% of total strategies and (2) Strategies for CG Social, Recreational, Personal Enhancement and Other Community Activities with 18 endorsements, 15.25% of total responses. These findings suggested that health-related and social, recreational, and personal enhancement strategies were the most commonly used by African-American CBI participants. Intercoder agreement was assessed across two one-month coding intervals, each of which contained 5 coding sessions. Mean percent of agreement and Kappa were .94 and .93, respectively. Both reliability measures fell within the highly acceptable range. Coder drift was minimal across the two time intervals. Future research will evaluate the psychometric properties of the DCSI-A with a larger sample of African-American CGs undergoing CBI.

